# 

**Published:** 1838-07-01

**Authors:** 


### CONTENTS

OF THE

### MEDICO-CHIRURGICAL REVIEW,

No. LVII. JULY 1, 1838,

[BEING No. XVII. OF A DECENNIAL SERIES.]

### REVIEWS.

I.

DISEASES OF THE RECTUM.

1. A Treatise on the Malformations, Injuries, and Diseases of the Rectum and Anus. By GEORGE BUSHE, M.D. .. } 1

2. On the Diseases of the Rectum. By JAMES SYME, F.R.S.E... .. }

   1. Abscess near the Anus, and Fistula in Ano .. 1

      A. Abscesses independent of Disease of the Rectum .. 1

      B. Abscesses arising from a Morbid Condition of the Rectum .. 4

   2. Contraction of the Anus .. 17

   3. Stricture of the Rectum .. 18

      A. Spasmodic Stricture of the Rectum .. 18

      B. Organic Stricture of the Rectum .. 19

      C. Simple Stricture of the Rectum .. 21

      D. Malignant Stricture, or Carcinomatous Degeneration of the Rectum .. 31

   Polypus of the Rectum .. 35

II.

ANATOMY OF THE TEETH.

1. Anatomie du Systeme Dentaire, considérée dans l'Homme et les Animaux. Par PH. FR. BLANDIN .. } 38

2. The Works of JOHN HUNTER, F.R.S. With Notes. Vol. II... .. }

   1. History of the Anatomy of the Teeth .. 39

   2. The Teeth in general .. 40

   3. Of the Human Teeth .. 41

      A. The Organization of the Teeth .. 42

      B. Development of the Teeth .. 46

      C. Growth of the Fang .. 50

      D. Formation of the permanent Teeth .. 51

   4. Teeth of the Invertebrata .. 53

   5. Teeth of the Vertebrata .. 54

III.

ANIMAL MAGNETISM.

An Introduction to the Study of Animal Magnetism. By the Baron DUPOTET DE SENNEVOY .. 59

IV.

The Stomach in its Morbid States, &c. &c. By LANGSTON PARKER, M.R.C. .. 97

   1. Anemia .. 98

   2. Confirmed Inflammatory Affections of Stomach .. 101

   3. Morbid Sensibility of the Nerves of the Stomach .. 102

   4. On the Influence of the Stomach upon other Organs .. 106

V.

Preliminary Communications regarding an Enquiry into Artificial Digestion. By Prof. Dr. PURKINGE and PAPPENHEIM .. .. .. .. .. .. .. .. .. .. .. .. .. 114
1. On the Influence of Galvanism in Artificial Digestion .. .. .. .. 114
2. On the Effects of certain Mechanical Agents in the Artificial Digestion of Coagulated Albumen .. .. .. .. .. .. .. .. .. .. .. .. .. 120

VI.

A Treatise on the Nature, Symptoms, Causes, and Treatment of Insanity, &c. &c. By Sir W. C. ELLIS, M.D... .. .. .. .. .. .. .. .. .. .. .. .. .. 122

VII.

The Nature and Treatment of Diseases of the Ear. By Dr. WILLIAM KRAMER .. .. 129

VIII.

On Reflex Motions. By Dr. H. W. VOLKMANN .. .. .. .. .. .. .. .. .. 142
1. State of Frogs after Decapitation .. .. .. .. .. .. .. .. .. .. .. 142
2. Simplest Phenomenon of Reflex Motions .. .. .. .. .. .. .. .. 143
3. Reflex Motions on dividing the Spinal Cord longitudinally.. .. .. .. 144
4. Reflex Motions have the Character of Determinateness .. .. .. .. 145
5. Extension of the Reflex Motions .. .. .. .. .. .. .. .. .. .. 145
6. On the Division of the Nerves proposed by M. Hall .. .. .. .. .. 149

IX.

FORENSIC MEDICINE.

A Medico-legal Treatise on Homicide by External Violence. By ALEXANDER WATSON, Esq. .. .. .. .. .. .. .. .. .. .. .. .. .. .. .. .. 152
1. General Remarks on Medical Jurisprudence.. .. .. .. .. .. .. .. 152
2. General Remarks on Homicide and Death by Violence .. .. .. .. 155
3. Medico-legal Definitions of Wounds .. .. .. .. .. .. .. .. .. .. 155
4. Of Homicide by Injuries of the Nervous System.. .. .. .. .. .. .. 156
5. Of Homicide by Injuries of the Circulating System .. .. .. .. .. .. 160
6. Of Homicide by Injuries of the Respiratory System .. .. .. .. .. .. 162
7. Of Homicide by Injuries of the Nutritive System .. .. .. .. .. .. 164

X.

On Diseases of the Bladder. By WILLIAM COULSON, Surgeon .. .. .. .. .. .. 166
1. Irritability of the Bladder .. .. .. .. .. .. .. .. .. .. .. .. 166
2. Paralysis of the Bladder.. .. .. .. .. .. .. .. .. .. .. .. .. 172
3. Acute Inflammation of the Mucous Membrane of the Bladder .. .. .. 175
4. Subacute Inflammation of the Mucous Membrane of the Bladder .. .. 178
5. Acute Inflammation of the Muscular Structure of the Bladder .. .. .. 181
6. Chronic Inflammation of the Muscular Coat of the Bladder .. .. .. 183
7. Inflammation of the Peritoneal Coat of the Bladder .. .. .. .. .. .. 184
8. Fungus Hæmatodes and Cancer of the Bladder .. .. .. .. .. .. .. 185
9. Foreign Bodies in the Bladder; Operation for Stone .. .. .. .. .. 185

### PERISCOPE.

### Bibliographical Notices.

1. An Experimental Inquiry into the Physiology of Cutaneous Absorption, &c. By W. H. MADDEN, M.D. .. .. .. .. .. .. .. .. .. .. .. .. .. .. 187

2. Notes on the Medical Topography of Calcutta .. .. .. .. .. .. .. .. 190

3. The Reform of Criminals and of Prisons .. .. .. .. .. .. .. .. .. .. .. 191

4. Medical Portrait Gallery .. .. .. .. .. .. .. .. .. .. .. .. .. .. .. 192

1. Esculapius .. .. .. .. .. . .. .. .. .. .. .. .. .. 193

2. Albinus .. .. .. .. .. .. .. .. .. .. .. .. .. .. .. .. .. 193

3. Characteristic Sketch of Sir H. Halford. By one of the Editors.. .. .. 194

4. Ruysch .. .. .. .. .. .. .. .. .. .. .. .. .. .. .. .. 195

5. Akenside .. .. .. .. .. .. .. .. .. .. .. .. .. .. .. .. .. 196

6. Characteristics of Sir C. M. Clarke. By one of the Editors .. .. .. 196

5. Philosophy of Marriage. By Dr. RYAN .. .. .. .. .. .. .. .. .. .. .. 197

6. Observations on Sciatica, and the Baths of Bagneres and Barege. By Mr. CARMICHAEL .. .. .. .. .. .. .. .. .. .. .. .. .. .. .. .. .. 198

7. The Alternative; Disease and Premature Death—or Health and Long Life. By J. PINNEY, Esq. .. .. .. .. .. .. .. .. .. .. .. .. .. .. 201

8. Treatise on Hernia. By M. W. HILLES .. .. .. .. .. .. .. .. .. .. .. 203

9. Physical Education of Children. By Mr. SMILES .. .. .. .. .. .. . 204

10. Practical Treatise on Fractures. By Mr. LONSDALE .. .. .. .. .. .. 204

11. Experiments on the Gastric Juice. By Dr. BEAUMONT .. .. .. .. .. 205

12. History of British Birds. By Mr. YARRELL .. .. .. .. .. .. .. .. .. 207

13. History of British Reptiles. By Mr. BELL .. .. .. .. .. .. .. .. .. 207

14. A Series of Anatomical Plates. By Mr. JONES QUAIN .. .. .. .. .. .. 209

15. Velpeau's Anatomy of Regions. By Mr. H. HANCOCK .. .. .. .. .. .. 209

16. The Anatomy of the Regions, interested in the Surgical Operations performed on the Human Body. By J. LEBAUDY, M.D. .. .. .. .. .. .. .. .. 209

17. A Series of Anatomical Sketches and Diagrams. By Mr. THOMAS WORMALD .. 210

### Clinical Review, and Hospital Reports.

### GUY'S HOSPITAL.

1. On Abdominal Tumors .. .. .. .. .. .. .. .. .. .. .. .. .. .. .. .. 211
A. Acephalocyst Hydatids.. .. .. .. .. .. .. .. .. .. .. .. .. .. 212
B. Ovarian Tumors .. .. .. .. .. .. .. .. .. .. .. .. .. .. .. .. 219

### Spirit of the Foreign Periodicals, &c.

1. Occasional Existence of Animalculæ in the Human Blood .. .. .. .. .. .. 225

2. Remarks on Constitutional Hæmorrhages, with Cases. Use of the Sulphate of Soda as an Internal remedy .. .. .. .. .. .. .. .. .. .. .. .. .. .. 226

3. Remarks on Spasm of the Glottis. Influence of Enlargements of the Thymus Gland .. .. .. .. .. .. .. .. .. .. .. .. .. .. .. .. .. .. 228

4. On the Treatment of Phthisis .. .. .. .. .. .. .. .. .. .. .. .. .. .. 231

5. Two Cases of Genuine Gangrene of the Lungs .. .. .. .. .. .. .. .. 231

6. Quinine in Enlargements of the Spleen .. .. .. .. .. .. .. .. .. .. .. 233

7. On the Use of the solid Nitrate of Silver in Gonorrhœal and other Discharges in Females .. .. .. .. .. .. .. .. .. .. .. .. .. .. .. .. .. .. 233

8. M. Rayer on Diseases of the Kidneys .. .. .. .. .. .. .. .. .. .. .. 234

9. Illustrations of the Pathology of the Capsulæ Supra-renales. By M. RAYER .. 237

10. Case of Glanders in the Human Subject .. .. .. .. .. .. .. .. .. .. .. 239

11. On the Contagion of the Variola, during 1836, in Haischberg in Germany .. .. 240

12. Therapeutic Influence of Compression of the large Blood-vessels in Neuralgia, &c. 240

13. Utility of Ergot of Rye in Paralytic Affections .. .. .. .. .. .. .. .. 242

14. Researches on the Causes of Abortion. By Madame BOIVIN .. .. .. .. 242

15. Irregularities of the Bowels—a frequent Cause of Uterine Affections .. .. .. 244
16. Notice of the Hôtel Dieu of Paris .. .. .. .. .. .. .. .. .. .. .. 245
17. Wound of the Ear from the Insertion of a Knitting-needle—death .. .. .. 246
18. Blisters in unfavourable Cases of Erysipelas of the Scalp .. .. .. .. .. 246
19. On the Treatment of Fractures. By M. VELPEAU .. .. .. .. .. .. .. .. 247
20. M. GERDY on Bandages and other Surgical Apparatus .. .. .. .. .. .. 248
21. M. BOUCHERON on the Hair .. .. .. .. .. .. .. .. .. .. .. .. .. 249
22. Dr. CASPAR on the Duration of Human Life .. .. .. .. .. .. .. .. .. 250
23. On the Introduction of Air into the Veins, during Surgical Operations .. .. 253
24. On the Radical Cure of Herniæ, by Acupuncturation .. .. .. .. .. .. .. 256
25. Cases of Phlebitis after Amputation .. .. .. .. .. .. .. .. .. .. .. 259
26. On the Efficacy of Extension, Shampooing and Percussion in Muscular contractions. By Dr. RECAMIER .. .. .. .. .. .. .. .. .. .. .. 260
27. Proto-ioduret of Iron in Syphilis .. .. .. .. .. .. .. .. .. .. .. .. 264
28. Hooping-cough treated with Carbonate of Iron .. .. .. .. .. .. .. .. 265
29. On the Morbid Diatheses which have successively affected the Nations of Europe 265
30. On the Microscopical Examination of the Urine .. .. .. .. .. .. .. .. 267
31. On the Relations in point of Numbers, of the Male and Female-sex .. .. .. 269
32. Medico-legal Considerations of the most frequent Causes of Death .. .. .. 270

### Miscellanies.

1. Results of Tapping the Head in Nineteen Cases of Hydrocephalus. By J. T. CONQUEST, M.D. .. .. .. .. .. .. .. .. .. .. .. .. .. .. 273

2. The Medical Topography of Cove. By D. H. SCOTT, M.D. .. .. .. .. .. .. 276

3. Bronchotomy .. .. .. .. .. .. .. .. .. .. .. .. .. .. .. .. .. 278

4. Thames' Improvement Company .. .. .. .. .. .. .. .. .. .. .. .. 280

5. Bronchocele removal by Croton Oil .. .. .. .. .. .. .. .. .. .. .. 281

6. Climate and Diseases of Swan River. By Dr. MILLIGAN .. .. .. .. .. .. 281

7. Bite of a Cobra Capella—Venesection considered as the Cure .. .. .. .. 282

8. Land Scurvy—Nusserabad .. .. .. .. .. .. .. .. .. .. .. .. .. .. 282

9. Dicksonian " Finish " .. .. .. .. .. .. .. .. .. .. .. .. .. .. 283

10. Regulations and Instructions for the Naval Medical Officers .. .. .. .. 284

11. Animal Magnetism again .. .. .. .. .. .. .. .. .. .. .. .. .. .. 285

BIBLIOGRAPHICAL RECORD .. .. .. .. .. .. .. .. .. .. .. .. .. .. 286

### Extra-Limites.

### HOSPITAL REPORTS.

1. Account of some Remarkable Cases of Dropsy, with the Necroscopic Appearances. By Sir DAVID J. H. DICKSON, M.D. .. .. .. .. .. .. .. .. .. .. 289
2. Surgical Report of the Dumfries and Galloway Royal Infirmary, for the year 1836-37 .. .. .. .. .. .. .. .. .. .. .. .. .. .. .. .. .. 293

### REGIMENTAL HOSPITAL, BANGALORE.

3. Purulent Discharges from the Bladder and Rectum in Hepatic Diseases, &c. By J. MOUAT, Esq. .. .. .. .. .. .. .. .. .. .. .. .. .. .. .. 306
A. Appendix .. .. .. .. .. .. .. .. .. .. .. .. .. .. .. .. 311

### CALCUTTA NATIVE HOSPITAL.

4. Return of Cases of Hydrocele treated at the Native Hospital, Calcutta, by J. R. MARTIN, Esq... .. .. .. .. .. .. .. .. .. .. .. .. .. .. .. 319
5. Clinical Contributions from Addenbrooke's Hospital, Cambridge. By HENRY BOND, M.D. .. .. .. .. .. .. .. .. .. .. .. .. .. .. .. .. 320
A. Diseases of the Pulmonary Organs .. .. .. .. .. .. .. .. .. 322
B. Diseases of the Heart .. .. .. .. .. .. .. .. .. .. .. .. .. 339

